# Predictors of sudden cardiac death in atrial fibrillation: The Atherosclerosis Risk in Communities (ARIC) study

**DOI:** 10.1371/journal.pone.0187659

**Published:** 2017-11-08

**Authors:** Ryan J. Koene, Faye L. Norby, Ankit Maheshwari, Mary R. Rooney, Elsayed Z. Soliman, Alvaro Alonso, Lin Y. Chen

**Affiliations:** 1 Cardiovascular Division, Department of Medicine, University of Minnesota Medical School, Minneapolis, Minnesota, United States of America; 2 Division of Epidemiology and Community Health, School of Public Health, University of Minnesota, Minneapolis, Minnesota, United States of America; 3 Epidemiological Cardiology Research Center (EPICARE), Department of Epidemiology and Prevention, and Department of Internal Medicine-Cardiology, Wake Forest School of Medicine, Winston Salem, North Carolina, United States of America; 4 Department of Epidemiology, Rollins School of Public Health, Emory University, Atlanta, Georgia, United States of America; Pennsylvania State University, UNITED STATES

## Abstract

We previously reported that incident atrial fibrillation (AF) is associated with an increased risk of sudden cardiac death (SCD) in the general population. We now aimed to identify predictors of SCD in persons with AF from the Atherosclerosis Risk in Communities (ARIC) study, a community-based cohort study. We included all participants who attended visit 1 (1987–89) and had no prior AF (n = 14,836). Incident AF was identified from study electrocardiograms and hospitalization discharge codes through 2012. SCD was physician-adjudicated. We used cause-specific Cox proportional hazards models, followed by stepwise selection (backwards elimination, removing all variables with p>0.10) to identify predictors of SCD in participants with AF. AF occurred in 2321 (15.6%) participants (age 45–64 years, 58% male, 18% black). Over a median of 3.3 years, SCD occurred in 110 of those with AF (4.7%). Predictors of SCD in AF included higher age, body mass index (BMI), coronary heart disease, hypertension, diabetes, current smoker, left ventricular hypertrophy, increased heart rate, and decreased albumin. Predictors associated only with SCD and not other cardiovascular (CV) death included increased BMI (HR per 5-unit increase, 1.15, 95% CI, 0.97–1.36, p = 0.10), increased heart rate (HR per SD increase, 1.18, 95% CI 0.99–1.41, p = 0.07), and low albumin (HR per SD decrease 1.23, 95% CI 1.02–1.48, p = 0.03). In the ARIC study, predictors of SCD in AF that are not associated with non-sudden CV death included increased BMI, increased heart rate, and low albumin. Further research to confirm these findings in larger community-based cohorts and to elucidate the underlying mechanisms to facilitate prevention is warranted.

## Introduction

Atrial fibrillation (AF) is the most common chronic arrhythmia in the United States, with an estimated prevalence of 5.2 million in 2010 [1]. The detrimental complications of AF, including a markedly increased risk of stroke, heart failure, and overall mortality, impose a major public health burden [2,3]. More recently, we have recognized that AF is independently associated with a 2-fold increased risk of sudden cardiac death (SCD) in community-dwelling adults [4]. Little is known about the predictors of SCD in AF. Two recent post-hoc analyses of clinical trial data, one from the ENGAGE-TIMI 48 study (The Effective Anticoagulation with Factor Xa Next Generation in Atrial Fibrillation—Thrombolysis in Myocardial Infarction 48) and the other from the RE-LY trial (Randomized Evaluation of Long-Term Anticoagulant Therapy) [5,6], explored this knowledge gap. In these studies, SCD accounted for 32% and 22% of all AF-related deaths, respectively. Another study utilized data from a nation-wide health insurance database in Taiwan to assess this relationship [7]. Given limitations of these prior studies, including the highly selected participants inherent in randomized controlled trials, or in the Taiwan study, the lack of systematic adjudication of SCD, we extended this area of investigation among community-dwelling individuals that underwent rigorous adjudication of SCD. In the present study, we aimed to identify demographic or clinical factors that are associated with SCD in participants with AF in a community-based prospective cohort study—the Atherosclerosis Risk in Communities (ARIC) Study.

## Methods

### Study population

The ARIC Study is a prospective community-based cohort study designed to investigate the determinants of atherosclerosis and its clinical outcomes as well as variations in cardiovascular (CV) risk factors, medical care, and disease by race and sex [8]. Detailed methods have been previously published [8]. Briefly, from 1987 to 1989 (ARIC Study baseline), a total of 15,792 adults (55% women, 45–64 years of age) from four US communities (Forsyth County, North Carolina; Jackson, Mississippi; northwest suburbs of Minneapolis, Minnesota; and Washington County, Maryland) were enrolled and followed prospectively through examination 5 (2011–2013). Participants were mostly white in the Washington County and Minneapolis sites, exclusively black in Jackson, and a mix of both races in Forsyth County. In addition to the baseline examination (1987–1989), the ARIC Study has conducted 4 follow-up examinations (1990–1992, 1993–1995, 1996–1998, and 2011–2013), along with annual telephone calls to determine vital status and obtain information on hospitalizations from the previous year. Ongoing surveillance of local hospitals has simultaneously been used to identify hospitalizations of ARIC Study participants, and trained abstractors have collected information on discharge diagnoses. The ARIC Study was approved by the institutional review board at each participating center, and written informed consent was obtained from all participants. A full list of participating institutional review boards can be found in the supporting information (S1 Table).

**Fig 1:**
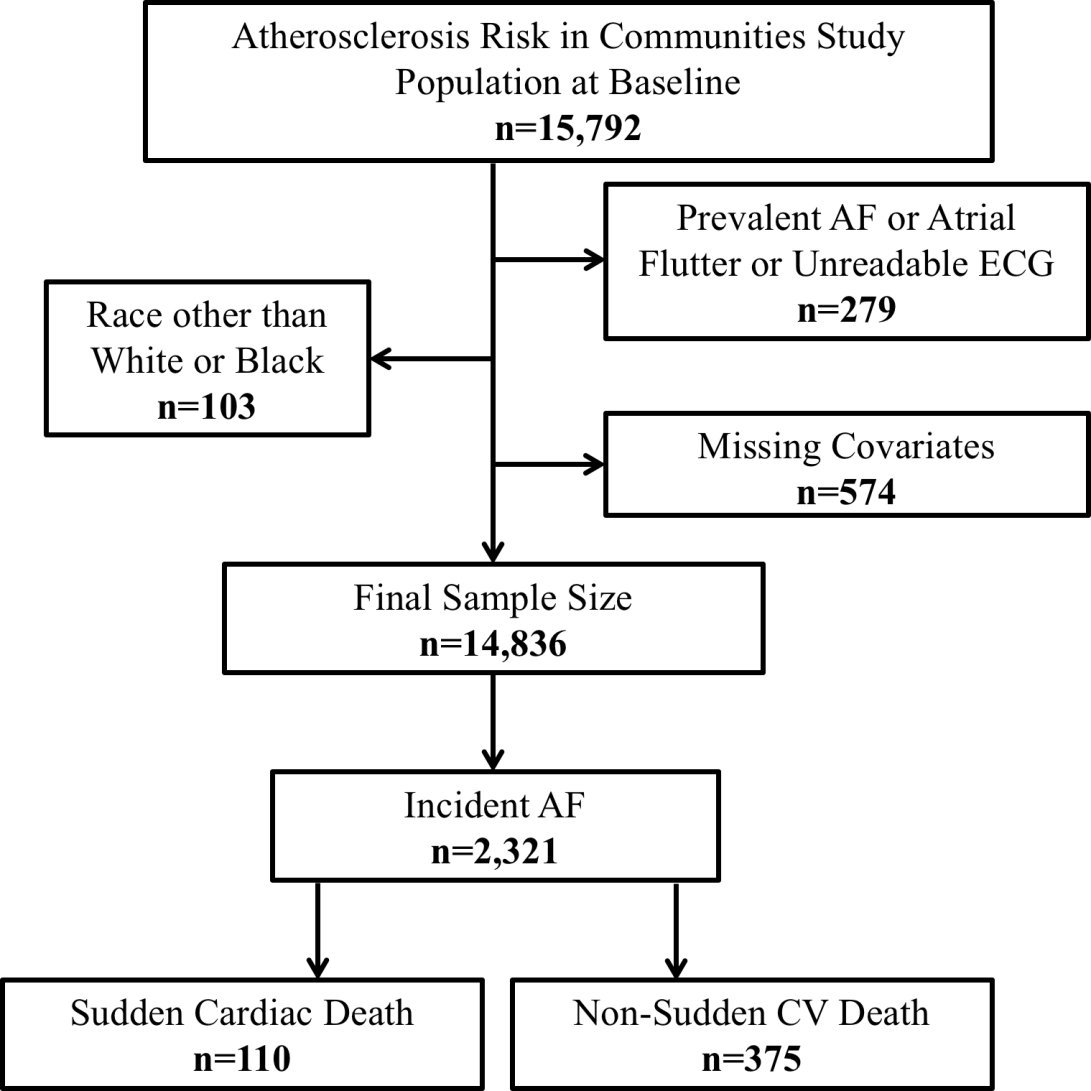
Flow chart of participants in the atherosclerosis risk in communities study, 1987, and outcomes of those who developed incident atrial fibrillation. AF indicates atrial fibrillation; CV, cardiovascular; ECG, electrocardiogram.

Of the 15,792 ARIC participants at visit 1, we excluded 103 who were not white or black, 279 with prevalent AF or unreadable ECG, and 574 with missing covariates. After exclusions, the analysis cohort included 14,836 participants (Fig 1).

### Ascertainment of AF

For this study, we used visit 1 (1987–1989) as the baseline visit, and considered only incident AF occurring after that time. Individuals with AF identified at visit 1 were considered to have prevalent AF and were excluded from this analysis.

AF diagnoses were obtained from ECGs at study visits and hospital discharge records [9]. At each study exam, a 12-lead ECG was performed and transmitted electronically to the ARIC ECG reading center at EPICARE (Wake Forest School of Medicine, Winston-Salem, NC) for review and analysis using the GE Marquette 12-SL program (GE Marquette, Milwaukee, WI). The presence of AF or atrial flutter in the ECG was identified by a computer algorithm and confirmed by a cardiologist. If ECGs had any rhythm disorder other than AF, they were over-read by a cardiologist to reduce any missed episodes of AF. Information on hospitalizations during follow-up was obtained from annual follow-up calls and surveillance of local hospitals, with hospital discharge diagnoses codes collected by trained abstractors. AF during follow-up was considered to be present if the International Classification of Disease 9th revision Clinical Modification [ICD-9-CM] codes 427.31 or 427.32 were listed in any given hospitalization. AF cases associated with open cardiac surgery were not included in this study. Previous studies in the ARIC cohort and other populations have demonstrated adequate validity of discharge codes for the identification of AF [9,10].

### Outcomes ascertainment

All ARIC Study participants were contacted annually by phone, and all hospitalizations and deaths in the previous year were identified [11]. Comprehensive data were gathered on CV events and deaths from hospital records; interviews with physicians, next of kin, and/or witnesses; death certificates; and autopsy reports, where available. Causes of death were adjudicated by the ARIC Study events committees. An independent review of coronary heart disease (CHD) deaths [12] was conducted to identify SCD events. The primary outcome, SCD, was defined in the ARIC Study as a sudden pulseless condition presumed to be due to a ventricular tachyarrhythmia in a previously stable individual without evidence of a noncardiac cause of cardiac arrest. All SCD cases occurred outside the hospital or in the emergency department, and the individuals could not have a life-threatening noncardiac comorbidity or be under hospice care. For unwitnessed deaths, the participant must have been seen within 24 hours of the arrest in a stable condition and without evidence of a noncardiac cause of cardiac arrest.

In the ARIC Study, all CHD deaths that occurred through 2012, were reviewed by a panel of 5 physicians to identify SCD events. Each event was independently adjudicated by 2 physicians. If there was disagreement, a third investigator reviewed the event to provide final classification. After review of available data, CHD deaths were classified as definite sudden arrhythmic death, possible sudden arrhythmic death, not sudden arrhythmic death, or unclassifiable [4,11,13]. For the present analysis, SCD was defined as definite or possible sudden arrhythmic deaths.

### Measurement of other covariates

Covariates that have been reported to be associated with an increased risk of SCD in the broader population of typically non-AF persons were used for this study [5,6,14–17]. We used covariate measurements from the study visit immediately preceding incident AF diagnosis, thus from either visit 1, 2, 3, or 4. Definitions of the covariates are detailed in the Supporting Methods.

### Statistical analysis

We report means with standard deviations (SDs) or medians and interquartile ranges (IQR) for continuous variables and counts with percentages for categorical variables. To evaluate the association of candidate variables with SCD, non-sudden CV death, and non-cardiovascular death, we used a cause-specific hazard model—a specific method used in competing risks analysis—which can be estimated with a Cox regression hazards model [18]. In addition, we performed a sensitivity analysis by fitting the Fine-Gray model, a proportional hazards model for the subdistribution of competing risks [19]. Except for potassium (taken at visit 1 only), ankle brachial index (visit 1 and 4), and albumin (visit 1 only), candidate variables were assessed at the visit just before AF ascertainment. First, we included all variables in the multivariable model. Next, we used a stepwise selection method (backwards elimination, removing all variables with a p-value >0.10) to fit the most parsimonious model for predicting SCD. Quadratic terms were evaluated for continuous variables. We performed the same analyses for non-sudden CV death. The proportional hazards assumption was assessed with scaled Schoenfeld residuals for both graphical and numerical tests, time interaction terms, and inspection of log negative log survival curves. Modeling assumptions were not violated in any model. Statistical analysis was performed using SAS version 9.2 (SAS Institute Inc., Cary, NC). All *P* values reported were 2-sided.

## Results

During a median follow up of 23.3 years, 2321 (16%) developed incident AF. From this subset of individuals, we identified 110 (4.7%) cases of SCD over a median follow up of 3.3 years from the time of incident AF. A total of 375 non-sudden CV deaths occurred (Fig 1).

Table 1 shows characteristics of ARIC Study participants at the study visit preceding AF ascertainment, stratified by subsequent SCD occurrence. Mean age ± SD was 69.9±7.6 years in the SCD group versus 72.4±7.9 years in those without SCD. Blacks comprised 26% of those with SCD but only 18% of the overall AF population. Comorbid medical conditions were more prevalent in those with SCD than those without, including increased body mass index (BMI), CHD, heart failure, hypertension, diabetes, current smoking, LVH, and ankle brachial index<0.9. Those who developed SCD were also more likely to be on cardiac medications.

**Table 1:** Characteristics of ARIC study participants at visit preceding AF diagnosis, stratified by mode of death.

		AF (n = 2321)		
Variables	All (n = 2321)	SCD (n = 110)	Non-sudden CV death (n = 375)	Non-CV deaths (n = 786)
Age, years (SD)	72.3 (7.9)	69.9 (7.6)	71.9 (7.5)	71.5 (7.5)
Men	1244 (54)	66 (60)	195 (52)	482 (61)
Black race	426 (18)	29 (26)	102 (27)	139 (18)
Body mass index, kg/m^2^ (SD)	29.6 (6.1)	31.0 (7.2)	29.6 (6.3)	29.6 (6.3)
Coronary heart disease	378 (16)	42 (38)	91 (24)	138 (18)
Heart failure	105 (5)	9 (8)	37 (10)	38 (5)
Hypertension	1358 (59)	81 (74)	274 (73)	467 (59)
Diabetes	563 (24)	50 (45)	126 (34)	215 (27)
Current smoker	504 (22)	30 (27)	93 (25)	225 (29)
eGFR, ml/min/1.73m^2^ (SD)	83.6 (18.9)	81.2 (22.1)	79.3 (21.0)	82.3 (20.4)
LVH by ECG criteria	107 (5)	13 (12)	33 (9)	30 (4)
Heart rate, bpm (SD)	64.3 (11.9)	67 (13.0)	65.4 (12.5)	66.6 (12.8)
QTc interval, ms (SD)	424.4 (25.2)	428 (26.5)	429.3 (28.8)	425.2 (27.2)
HDL, g/dL (SD)	47.2 (16.2)	43.1 (12.3)	46.5 (16.8)	45.7 (16.0)
Beta blockers	158 (7)	16 (15)	40 (11)	68 (9)
Anti-arrhythmics	44 (2)	3 (3)	16 (4)	16 (2)
Digoxin	179 (8)	18 (16)	59 (16)	55 (7)
Potassium level (SD)	4.2 (0.4)	4.2 (0.4)	4.2 (0.5)	4.2 (0.5)
ABI ≤0.9	156 (7)	11 (10)	32 (9)	66 (8)
Albumin, g/dL (SD)	3.9 (0.3)	3.8 (0.3)	3.8 (0.3)	3.8 (0.3)

Data shown are n (%) unless otherwise indicated. ABI indicates ankle brachial index; AF, atrial fibrillation; ARIC, Atherosclerosis Risk in Communities; bpm, beats per minute; CV, cardiovascular; dL, deciliter; ECG, electrocardiogram; g, grams; HDL, high density lipoprotein cholesterol; HR, heart rate; LVH, left ventricular hypertrophy; ms, milliseconds; QTc, corrected QT; SCD, sudden cardiac death; SD, standard deviation.

### Predictors of SCD and non-sudden CV death using cause-specific hazards models

Table 2 lists the candidate variables, showing multivariable Cox proportional hazards ratios (HR) for their association with SCD, prior to backwards elimination. In the multivariable cause-specific method—accounting for non-sudden CV deaths and non-cardiovascular deaths—predictors of SCD in AF after backwards elimination included higher age, increased BMI, CHD, hypertension, diabetes, current smoker, LVH, increased heart rate, and decreased albumin (Table 3).

**Table 2:** Multivariable cox proportional hazard ratios (95% confidence interval)* for SCD and non-sudden CV death in ARIC participants with incident AF.

	# SCD = 110		# non-sudden CV deaths = 375	
Variables^†^	HR (95% CI)	P-value	HR (95% CI)	P-value
Age (per 5 years)	1.31 (1.09–1.59)	0.005	1.29 (1.16–1.42)	<0.0001
Men	1.39 (0.89–2.17)	0.15	1.01 (0.80–1.28)	0.92
Black race	1.53 (0.94–2.49)	0.09	1.75 (1.36–2.25)	<0.0001
Body mass index, kg/m^2^ (per 5 unit increase)	1.16 (0.98–1.39)	0.09	0.97 (0.88–1.07)	0.48
Coronary heart disease	3.07 (2.01–4.69)	<0.0001	1.64 (1.27–2.12)	0.0001
Heart failure	0.94 (0.45–1.96)	0.87	1.54 (1.06–2.25)	0.02
Hypertension	1.53 (0.98–2.40)	0.06	1.70 (1.34–2.16)	<0.0001
Diabetes	2.28 (1.47–3.52)	0.0002	1.63 (1.27–2.07)	<0.0001
Current smoker	1.87 (1.17–2.99)	0.009	1.51 (1.17–1.95)	0.002
eGFR, ml/min/1.73m^2^ (per SD increase)	1.14 (0.94–1.38)	0.19	1.20 (1.08–1.32)	0.0004
LVH by ECG criteria	2.10 (1.11–3.98)	0.02	1.39 (0.96–2.03)	0.08
Heart rate, bpm (per SD increase)	1.17 (0.97–1.41)	0.10	1.06 (0.95–1.17)	0.30
QTc interval, ms (per SD increase)	1.07 (0.49–2.35)	0.86	1.59 (1.10–2.31)	0.01
HDL, g/dL (per SD decrease)	1.06 (0.83–1.36)	0.62	1.00 (0.89–1.13)	0.98
Beta blockers	1.35 (0.76–2.42)	0.31	1.04 (0.73–1.47)	0.84
Anti-arrhythmics	0.69 (0.21–2.33)	0.55	1.05 (0.61–1.81)	0.85
Digoxin	1.43 (0.81–2.54)	0.22	1.30 (0.95–1.78)	0.10
Potassium level (per SD increase)	0.91 (0.76–1.09)	0.29	1.04 (0.95–1.15)	0.41
ABI ≤0.9	0.93 (0.46–1.85)	0.83	0.92 (0.62–1.35)	0.65
Albumin, g/dL (per SD decrease)	1.20 (0.99–1.46)	0.07	1.05 (0.94–1.17)	0.39

ABI indicates ankle brachial index; AF, atrial fibrillation; ARIC, Atherosclerosis Risk in Communities; bpm, beats per minute; dL, deciliter; ECG, electrocardiogram; g, grams; HDL, high density lipoprotein cholesterol; HR, heart rate; LVH, left ventricular hypertrophy; ms, milliseconds; QTc, corrected QT; SCD, sudden cardiac death; SD, standard deviation.

* Cause-specific analysis

† Variables measured at visit prior to AF diagnosis

**Table 3:** Parsimonious multivariable cox proportional hazard model* for prediction^†^ of SCD and non-sudden CV death in participants with AF in the ARIC study.

	# SCD = 110		# non-sudden CV	
			deaths = 375	
Variables	HR (95% CI)	P-value	HR (95% CI)	P-value
Age (per 5 years)	1.32 (1.11–1.58)	0.002	1.28 (1.16–1.41)	<0.0001
BMI (per 5 unit increase)	1.15 (0.97–1.36)	0.10		
Coronary heart disease	3.27 (2.20–4.87)	<0.0001	1.64 (1.28–2.10)	<0.0001
Hypertension	1.69 (1.10–2.61)	0.02	1.68 (1.32–2.12)	<0.0001
Diabetes	2.41 (1.59–3.65)	<0.0001	1.61 (1.28–2.01)	<0.0001
Current smoker	1.85 (1.18–2.90)	0.007	1.57 (1.24–2.00)	0.0002
LVH by ECG criteria	2.30 (1.26–4.21)	0.007	1.41 (0.97–2.04)	0.07
Heart rate, bpm (per SD increase)	1.18 (0.99–1.41)	0.07		
Albumin (per SD decrease)	1.23 (1.02–1.48)	0.03		
Race (black)			1.76 (1.38–2.23)	<0.0001
Heart failure			1.58 (1.09–2.28)	0.02
Digoxin			1.33 (0.99–1.79)	0.06
eGFR (per SD decrease)			1.21 (1.10–1.33)	0.0002
QTc interval (per SD increase)			1.64 (1.15–2.35)	0.006

AF indicates atrial fibrillation; ARIC, Atherosclerosis Risk in Communities; BMI, body mass index; bpm, beats per minute; CV, cardiovascular; ECG, electrocardiogram; eGFR, estimated glomerular filtration rate; HR, hazard ratio; LVH, left ventricular hypertrophy; QTc, corrected QT interval; SCD, sudden cardiac death; SD, standard deviation.

* Cause-specific analysis

† Significant predictors were obtained using backwards elimination (p<0.10) of the candidate predictor variables.

Table 2 also shows the multivariable HR of candidate variables for non-sudden CV death, prior to backwards elimination. After backwards elimination, predictors of non-sudden CV death in AF included higher age, black race, CHD, heart failure, hypertension, diabetes, current smoker, LVH, digoxin use, decreased eGFR, and prolonged QTc interval (Table 3).

The predictors significant for SCD but not non-sudden CV death were increased BMI (HR, 1.15, 95% CI, 0.97–1.36, p = 0.10) increased heart rate (HR, 1.18, 95% CI 0.99–1.41, p = 0.07), and low albumin (HR 1.23, 95% CI 1.02–1.48, p = 0.03) (Table 3).

### Predictors of SCD and non-sudden CV death using the Fine-Gray method

S2 Table shows the multivariable HR of candidate variables for SCD, prior to backwards elimination. After backwards elimination, predictors of SCD in AF using the Fine-Gray method included higher age, increased BMI, CHD, hypertension, diabetes, current smoker, LVH, and decreased albumin (S3 Table). S2 Table shows the multivariable HR of candidate variables for non-sudden CV death, prior to backwards elimination. After backwards elimination, predictors of non-sudden CV death in AF using the Fine-Gray method included higher age, black race, CHD, heart failure, hypertension, diabetes, current smoker, LVH, digoxin, eGFR, and QTc interval (S3 Table).

The predictors significant for SCD but not non-sudden CV death were increased BMI (HR 1.14, 95% CI 0.97–1.36, p = 0.10) and low albumin (HR 1.19, 95% CI 1.00–1.42, p = 0.05) (S3 Table).

## Discussion

In this large community-based prospective cohort study, we found several clinical characteristics that were associated with SCD in participants who developed AF. CHD was the strongest predictor (>3-fold risk), followed by diabetes and LVH (>2-fold risk), and then higher age, increased BMI, hypertension, smoking, increased heart rate, and low albumin (<2-fold risk). Predictors specific to SCD but not non-sudden CV death included increased BMI, increased heart rate, and low albumin. These results were robust to a sensitivity analysis using the Fine-Gray method. To our knowledge, there has been only one other investigation—outside of post-hoc clinical trial data—to assess predictors of SCD in community-dwelling individuals with AF.

SCD is the most common mode of death in AF patients. In the post-hoc analysis from the RE-LY trial, which randomized over 18,000 AF patients to either warfarin or dabigatran, the most common mode of death was SCD, accounting for 22% of total deaths [6]. Similarly, the post-hoc analysis from the ENGAGE AF-TIMI 48 Trial, which randomized over 21,000 AF patients to either edoxaban or warfarin, found SCD to be the most common mode of death, comprising 32% of total deaths [5].

Our work advances current knowledge over published clinical trials (ENGAGE TIMI 48 and RELY), by extending investigation on this topic to community-dwelling individuals. Further, unlike the report by Chao et al. which did not account for the competing risk of death and that was based on a nation-wide insurance database that lacked adjudication of SCD, our analysis accounted for the competing risk of death and was based on systematic expert adjudication of SCD. The ENGAGE TIMI 48, RE-LY, and nation-wide study in Taiwan findings have several similarities and differences compared with our study. Similar to ENGAGE TIMI 48, we found age, CHD, LVH, higher heart rate, and increased BMI to predict SCD in AF, but unlike ENGAGE TIMI 48 we did not find an association with digoxin use, heart failure, male sex, non-use of beta blockers, or peripheral artery disease. Similar to RE-LY, we found CHD and diabetes to predict SCD in AF, but unlike RE-LY we did not find an association with heart failure or male sex. Similar to the Taiwan study, we found age, hypertension, and diabetes to predict SCD in AF, but unlike the Taiwan study we did not find an association with heart failure, chronic kidney disease, or peripheral artery disease. Heart failure is a potential risk factor for SCD in patients with AF as heart failure strongly predicts SCD and CV mortality across many population types [20]. RE-LY and ENGAGE TIMI 48 analyses found strong associations between heart failure with both SCD and non-sudden CV deaths in AF patients, but in our study it only predicted non-sudden CV death. The Taiwan study also found heart failure to predict SCD in AF. In our study, it is possible that the low prevalence of heart failure prior to AF ascertainment (i.e. 4.5%), along with relatively few SCD events, may have limited statistical detection.

In both ENGAGE TIMI 48 and our study, higher heart rate was a unique predictor of SCD and not of non-sudden CV death. A higher resting heart rate is a sign of sympathetic overactivity, which has been associated with SCD, usually resulting from ventricular arrhythmia [21,22]. It has also been suggested that ischemic episodes that trigger arrhythmias are more likely to do so at higher heart rates [22]. β-blockers are well known to reduce sudden arrhythmic death, which may in part be through their heart rate lowering effect [23].

The association between obesity and CV morbidity and mortality is well known [24]. Furthermore, the association between obesity and SCD has been described [17,25]. In our analysis, we found obesity, as measured by increased BMI, to be uniquely associated with SCD (but not with non-sudden CV death), whereas the ENGAGE TIMI 48 found obesity to be associated with both SCD and other CV deaths.

While low albumin is widely known for being a predictor of poor outcomes across a variety of clinical settings, in our study we found it to be a predictor of SCD but not non-sudden CV deaths [26]. A prior ARIC study observed an inverse association of albumin with cardiac death, but did not find an independent association with the incidence of non-fatal myocardial infarction [16]. A combined analysis from ARIC and the Cardiovascular Health Study also found low albumin to be an independent predictor of SCD [14]. These findings are in agreement with an analysis from the MRFIT study, which found low albumin levels to be more strongly associated with CHD mortality (the majority of deaths being SCD) than with nonfatal myocardial infarction [27]. Therefore, low albumin level appears to be a marker for SCD in both AF and non-AF populations; the underlying mechanisms, however, are unknown. Factors that may contribute to hypoalbuminemia include exogenous loss of albumin, albumin re-distribution, catabolism rate of proteins, and inflammation [28]; these factors may explain the association of hypoalbuminemia with SCD.

Several electrophysiological mechanisms for the association between AF and SCD have been postulated. For example, the rapid ventricular rate of AF may reduce ventricular refractoriness, thus increasing the excitable gap in an existent reentrant circuit, leading to life-threatening arrhythmias [29]. Also, the irregular rhythm of AF leads to short-long-short sequences that are intrinsically pro-arrhythmic [30]. AF-related adverse myocardial remodeling, tachycardia-induced cardiomyopathy, and impaired calcium handling are additional possible mechanisms [31,32]. Finally, the association could, in part, be influenced by clinical factors such as higher heart rate, obesity, low albumin, heart failure, and CHD [33]. Our study advances the field by identifying clinical risk factors for SCD, thus facilitating the potential discovery of novel SCD prevention strategies in patients with AF.

The strengths of our study include a prospective community-based investigation with meticulous physician-adjudication of SCD, the large number of incident AF cases, and extensive measurement of covariates. Several limitations should be noted. Predictor variables were single measurements, which may be limited by the precision and accuracy of the measuring instrument and any changes that may have occurred over time. There was a relatively small number of SCD and non-sudden CV death events in comparison to previously reported clinical trials, which may have limited our ability to identify additional predictors of SCD. In addition, due to the relatively small number of events, of the 3 predictors of SCD which are not predictors of nonsudden CV deaths (increased BMI, increased heart rate, and low albumin) only low albumin is statistically significant at the p = 0.05 level. Although our findings suggest that increased BMI, increased heart rate, and low albumin may be specific predictors of SCD, the underlying mechanisms remain unclear. Finally, underdetection of AF may have occurred in asymptomatic subjects and ascertainment of AF by hospital discharge codes could lead to false positives. However, with regard to the latter, adequate validity using hospital codes for the identification of incident AF has been demonstrated in the ARIC Study [9].

## Conclusion

In conclusion, our report—based on a large community-based cohort study—found that the predictors of SCD in persons with AF that are not associated with non-sudden CV deaths include increased BMI, increased heart rate, and low albumin. These findings will need to be confirmed in larger community-based cohorts with more SCD and non-sudden CV death events. Further research to elucidate the underlying mechanisms so as to facilitate discovery of novel SCD prevention strategies is warranted.

## Supporting information

S1 Supporting Methods (DOCX)

S1 Table Ethics Committee/Institutional Review Board(s) that approved this study. (DOCX)

S2 Table Proportional subdistribution hazard ratios (95% Confidence Interval) for SCD and Non-sudden CV death in ARIC participants with incident AF. (DOCX)

S3 Table Parsimonious proportional subdistribution hazard model for prediction† of SCD and non-sudden CV death in participants with AF in the ARIC study. (DOCX)

## References

[1] Colilla S, Crow A, Petkun W, Singer DE, Simon T, Liu X. Estimates of current and future incidence and prevalence of atrial fibrillation in the U.S. adult population. Am J Cardiol. 2013;112:1142–7. doi: 10.1016/j.amjcard.2013.05.063 23831166 10.1016/j.amjcard.2013.05.063

[2] Piccini JP, Hammill BG, Sinner MF, Jensen PN, Hernandez AF, Heckbert SR, et al Incidence and prevalence of atrial fibrillation and associated mortality among Medicare beneficiaries, 1993–2007. Circ Cardiovasc Qual Outcomes. 2012;5:85–93. doi: 10.1161/CIRCOUTCOMES.111.962688 22235070 10.1161/CIRCOUTCOMES.111.962688 PMC3332107

[3] Fauchier L, Villejoubert O, Clementy N, Bernard A, Pierre B, Angoulvant D, et al Causes of Death and Influencing Factors in Patients with Atrial Fibrillation. Am J Med. 2016;129:1278–1287. doi: 10.1016/j.amjmed.2016.06.045 27476087 10.1016/j.amjmed.2016.06.045

[4] Chen LY, Sotoodehnia N, Bůžková P, Lopez FL, Yee LM, Heckbert SR, et al Atrial fibrillation and the risk of sudden cardiac death: the atherosclerosis risk in communities study and cardiovascular health study. JAMA Intern Med. 2013;173:29–35. doi: 10.1001/2013.jamainternmed.744 23404043 10.1001/2013.jamainternmed.744 PMC3578214

[5] Eisen A, Ruff CT, Braunwald E, Nordio F, Corbalán R, Dalby A, et al Sudden Cardiac Death in Patients With Atrial Fibrillation: Insights From the ENGAGE AF-TIMI 48 Trial. J Am Heart Assoc. 2016;5 doi: 10.1161/JAHA.116.003735 27402235 10.1161/JAHA.116.003735 PMC5015407

[6] Marijon E, Le Heuzey JY, Connolly S, Yang S, Pogue J, Brueckmann M, et al Causes of death and influencing factors in patients with atrial fibrillation: a competing-risk analysis from the randomized evaluation of long-term anticoagulant therapy study. Circulation. 2013;128:2192–201. doi: 10.1161/CIRCULATIONAHA.112.000491 24016454 10.1161/CIRCULATIONAHA.112.000491

[7] Chao TF, Liu CJ, Tuan TC, Chen SJ, Chen TJ, Lip GYH, et al Risk and Prediction of Sudden Cardiac Death and Ventricular Arrhythmias for Patients with Atrial Fibrillation—A Nationwide Cohort Study. Sci Rep. 2017;7:46445 doi: 10.1038/srep46445 28422144 10.1038/srep46445 PMC5396069

[8] The Atherosclerosis Risk in Communities (ARIC) Study: design and objectives. The ARIC investigators. Am J Epidemiol. 1989;129:687–702. 2646917

[9] Alonso A, Agarwal SK, Soliman EZ, Ambrose M, Chamberlain AM, Prineas RJ, et al Incidence of atrial fibrillation in whites and African-Americans: the Atherosclerosis Risk in Communities (ARIC) study. Am Heart J. 2009;158:111–7. doi: 10.1016/j.ahj.2009.05.010 19540400 10.1016/j.ahj.2009.05.010 PMC2720573

[10] Jensen PN, Johnson K, Floyd J, Heckbert SR, Carnahan R, Dublin S. A systematic review of validated methods for identifying atrial fibrillation using administrative data. Pharmacoepidemiol Drug Saf. 2012;21 Suppl 1:141–7. doi: 10.1002/pds.2317 22262600 10.1002/pds.2317 PMC3674852

[11] Peacock JM, Ohira T, Post W, Sotoodehnia N, Rosamond W, Folsom AR. Serum magnesium and risk of sudden cardiac death in the Atherosclerosis Risk in Communities (ARIC) Study. Am Heart J. 2010;160:464–470. doi: 10.1016/j.ahj.2010.06.012 20826254 10.1016/j.ahj.2010.06.012 PMC2939007

[12] White AD, Folsom AR, Chambless LE, Sharret AR, Yang K, Conwill D, et al Community surveillance of coronary heart disease in the Atherosclerosis Risk in Communities (ARIC) Study: methods and initial two years’ experience. J Clin Epidemiol. 1996;49:223–33. 8606324 10.1016/0895-4356(95)00041-0

[13] Olson KA, Viera AJ, Soliman EZ, Crow RS, Rosamond WD. Long-term prognosis associated with J-point elevation in a large middle-aged biracial cohort: the ARIC study. Eur Heart J. 2011;32:3098–106. doi: 10.1093/eurheartj/ehr264 21785106 10.1093/eurheartj/ehr264 PMC3236999

[14] Deo R, Norby FL, Katz R, Sotoodehnia N, Adabag S, deFilippi CR, et al Development and Validation of a Sudden Cardiac Death Prediction Model for the General Population. Circulation. 2016;134:806–16. doi: 10.1161/CIRCULATIONAHA.116.023042 27542394 10.1161/CIRCULATIONAHA.116.023042 PMC5021600

[15] Friedman AN, Yu Z, Denski C, Tamez H, Wenger J, Thadhani R, et al Fatty Acids and Other Risk Factors for Sudden Cardiac Death in Patients Starting Hemodialysis. Am J Nephrol. 2013;38:12–18. doi: 10.1159/000351764 23816975 10.1159/000351764 PMC3856432

[16] Kucharska-Newton AM, Couper DJ, Pankow JS, Prineas RJ, Rea TD, Sotoodehnia N, et al Hemostasis, inflammation, and fatal and nonfatal coronary heart disease: long-term follow-up of the atherosclerosis risk in communities (ARIC) cohort. Arterioscler Thromb Vasc Biol. 2009;29:2182–90. doi: 10.1161/ATVBAHA.109.192740 19797708 10.1161/ATVBAHA.109.192740 PMC3057473

[17] Adabag S, Huxley RR, Lopez FL, Chen LY, Sotoodehnia N, Siscovick D, et al Obesity related risk of sudden cardiac death in the atherosclerosis risk in communities study. Heart. 2015;101:215–221. doi: 10.1136/heartjnl-2014-306238 25410499 10.1136/heartjnl-2014-306238 PMC4791977

[18] Austin PC, Lee DS, Fine JP. Introduction to the Analysis of Survival Data in the Presence of Competing Risks. Circulation. 2016;133:601–609. doi: 10.1161/CIRCULATIONAHA.115.017719 26858290 10.1161/CIRCULATIONAHA.115.017719 PMC4741409

[19] Fine JP, Gray RJ. A Proportional Hazards Model for the Subdistribution of a Competing Risk. J Am Stat Assoc. 1999;94:496–509. doi: 10.2307/2670170

[20] Epstein AE, DiMarco JP, Ellenbogen KA, Estes NAM, Freedman RA, Gettes LS, et al ACC/AHA/HRS 2008 Guidelines for Device-Based Therapy of Cardiac Rhythm Abnormalities: Executive Summary. Circulation. 2008;117:2820–2840. doi: 10.1161/CIRCUALTIONAHA.108.189742 18483207

[21] Jouven X, Empana J-P, Schwartz PJ, Desnos M, Courbon D, Ducimetière P. Heart-Rate Profile during Exercise as a Predictor of Sudden Death. N Engl J Med. 2005;352:1951–1958. doi: 10.1056/NEJMoa043012 15888695 10.1056/NEJMoa043012

[22] Fox K, Borer JS, Camm AJ, Danchin N, Ferrari R, Lopez Sendon JL, et al Resting Heart Rate in Cardiovascular Disease. J Am Coll Cardiol. 2007;50:823–830. doi: 10.1016/j.jacc.2007.04.079 17719466 10.1016/j.jacc.2007.04.079

[23] Al-Gobari M, Khatib C El, Pillon F, Gueyffier F. Beta-blockers for the prevention of sudden cardiac death in heart failure patients: a meta-analysis of randomized controlled trials. BMC Cardiovasc Disord. 2013;13:52 doi: 10.1186/1471-2261-13-52 23848972 10.1186/1471-2261-13-52 PMC3716800

[24] Poirier P, Giles TD, Bray GA, Hong Y, Stern JS, Pi-Sunyer FX, et al Obesity and cardiovascular disease: pathophysiology, evaluation, and effect of weight loss: an update of the 1997 American Heart Association Scientific Statement on Obesity and Heart Disease from the Obesity Committee of the Council on Nutrition, Physical. Circulation. 2006;113:898–918. doi: 10.1161/CIRCULATIONAHA.106.171016 16380542 10.1161/CIRCULATIONAHA.106.171016

[25] Soliman EZ, Prineas RJ, Case LD, Russell G, Rosamond W, Rea T, et al Electrocardiographic and clinical predictors separating atherosclerotic sudden cardiac death from incident coronary heart disease. Heart. 2011;97:1597–1601. doi: 10.1136/hrt.2010.215871 21775508 10.1136/hrt.2010.215871 PMC3638973

[26] Goldwasser P, Feldman J. Association of serum albumin and mortality risk. J Clin Epidemiol. 1997;50:693–703. 9250267 10.1016/s0895-4356(97)00015-2

[27] Kuller LH, Eichner JE, Orchard TJ, Grandits GA, McCallum L, Tracy RP. The relation between serum albumin levels and risk of coronary heart disease in the Multiple Risk Factor Intervention Trial. Am J Epidemiol. 1991;134:1266–77. 1755441 10.1093/oxfordjournals.aje.a116030

[28] Gopal DM, Kalogeropoulos AP, Georgiopoulou V, Tang WWH, Methvin A, Smith AL, et al Serum albumin concentration and heart failure risk The Health, Aging, and Body Composition Study. Am Heart J. 2010;160:279–85. doi: 10.1016/j.ahj.2010.05.022 20691833 10.1016/j.ahj.2010.05.022 PMC2919495

[29] Denes P, Wu D, Dhingra R, Pietras RJ, Rosen KM. The effects of cycle length on cardiac refractory periods in man. Circulation. 1974;49:32–41. 4271710 10.1161/01.cir.49.1.32

[30] Denker S, Lehmann M, Mahmud R, Gilbert C, Akhtar M. Facilitation of ventricular tachycardia induction with abrupt changes in ventricular cycle length. Am J Cardiol. 1984;53:508–15. 6198893 10.1016/0002-9149(84)90022-5

[31] Ling L, Khammy O, Byrne M, Amirahmadi F, Foster A, Li G, et al Irregular rhythm adversely influences calcium handling in ventricular myocardium: implications for the interaction between heart failure and atrial fibrillation. Circ Heart Fail. 2012;5:786–93. doi: 10.1161/CIRCHEARTFAILURE.112.968321 23014130 10.1161/CIRCHEARTFAILURE.112.968321

[32] Piccini JP, Daubert JP. Atrial fibrillation and sudden cardiac death: is heart failure the middleman? JACC Heart Fail. 2014;2:228–9. doi: 10.1016/j.jchf.2014.03.004 24952688 10.1016/j.jchf.2014.03.004

[33] Soliman EZ, Safford MM, Muntner P, Khodneva Y, Dawood FZ, Zakai NA, et al Atrial fibrillation and the risk of myocardial infarction. JAMA Intern Med. 2014;174:107–14. doi: 10.1001/jamainternmed.2013.11912 24190540 10.1001/jamainternmed.2013.11912 PMC4115282

