# Supplementary material for: Predictors of sudden cardiac death in atrial fibrillation: The Atherosclerosis Risk in Communities (ARIC) study
Source: PLoS One. 2017 Nov 8;12(11):e0187659. doi: 10.1371/journal.pone.0187659 (PMC5678684; doi:10.1371/journal.pone.0187659)
Supplement: S1 Supporting Methods — (DOCX) [file pone.0187659.s001.docx]

**Supporting Information Online Content**

**Supplementary Methods**

**Supplementary References**

**Supplementary Tables**

This supplementary material has been provided by the authors to give readers additional information about their work.

**Supplementary Methods**

At each visit, ARIC Study participants underwent a standardized medical history and examination that included interviews, fasting blood samples, measurements of anthropometry, 12-lead ECG, among other tests [1]. Trained interviewers ascertained basic demographic data, medical history, and personal habits (e.g. smoking, medication use, etc.). Participants were asked to bring containers of all current medications to the visit. Details about medication use were previously described [1]. Participants provided information on education, sex, race, smoking status, and prevalence of CHD. Body mass index (BMI) was calculated as the weight in kilograms divided by the height in meters squared. Blood pressure was measured with a random-zero sphygmomanometer after 5 minutes of rest in the siting position, and averaged over 2 measurements. Hypertension was defined as use of medication to treat high blood pressure, systolic blood pressure ≥140mm Hg, or diastolic blood pressure ≥90 mm Hg. Diabetes was defined as a fasting glucose level of ≥126 mg/dl, non-fasting glucose of ≥200 mg/dl, treatment of diabetes, or a self-reported medical diagnosis of diabetes. Prevalent CHD at visit 1 was defined as physician-diagnosed CHD or by the presence of previous myocardial infarction on ECG, while CHD occurring after visit 1 was adjudicated by the ARIC Morbidity and Mortality Classification Committee using information obtained from follow-up calls, hospitalization records, and study visit ECGs as previously published [2]. Heart failure at visit 1 was defined as the reported use of medications to treat heart failure in the previous 2 weeks or the presence of heart failure according to Gothenburg criteria. Heart failure occurring after visit 1 was defined as the presence of ICD-9-CM code 428 in any hospitalization during follow-up [3]. Left ventricular hypertrophy (LVH) was diagnosed using ECG criteria as described previously [4]. Serum creatinine was assessed in 99% of subjects at the initial visit [5]. Estimated glomerular filtration rate (eGFR) based on creatinine (eGFRcreat) was calculated from the CKD Epidemiology Collaboration equation for creatinine [6]. Potassium was only measured at visits 1 and 2, ankle-brachial index at visit 1 (96.4% of participants) and for selected individuals at either visit 3 or 4 [7], and albumin at visit 1.

**Supplementary References**

1. The Atherosclerosis Risk in Communities (ARIC) Study: design and objectives. The ARIC investigators. *Am J Epidemiol* [Internet]. 1989;129:687–702. Available from: http://www.ncbi.nlm.nih.gov/pubmed/2646917

2. White AD, Folsom AR, Chambless LE, Sharret AR, Yang K, Conwill D, et al. Community surveillance of coronary heart disease in the Atherosclerosis Risk in Communities (ARIC) Study: methods and initial two years’ experience. *J Clin Epidemiol* [Internet]. 1996 [cited 2015 Apr 2];49:223–33. Available from: http://www.ncbi.nlm.nih.gov/pubmed/8606324

3. Loehr LR, Rosamond WD, Chang PP, Folsom AR, Chambless LE. Heart failure incidence and survival (from the Atherosclerosis Risk in Communities study). *Am J Cardiol* [Internet]. 2008 [cited 2015 Apr 2];101:1016–22. Available from: http://www.ncbi.nlm.nih.gov/pubmed/18359324

4. Okwuosa TM, Soliman EZ, Lopez F, Williams KA, Alonso A, Ferdinand KC. Left ventricular hypertrophy and cardiovascular disease risk prediction and reclassification in blacks and whites: the Atherosclerosis Risk in Communities Study. *Am Heart J* [Internet]. 2015 [cited 2017 Jan 15];169:155–61.e5. Available from: http://www.ncbi.nlm.nih.gov/pubmed/25497261

5. Weiner DE, Krassilnikova M, Tighiouart H, Salem DN, Levey AS, Sarnak MJ. CKD classification based on estimated GFR over three years and subsequent cardiac and mortality outcomes: a cohort study. *BMC Nephrol* [Internet]. 2009 [cited 2015 Apr 2];10:26. Available from: http://www.biomedcentral.com/1471-2369/10/26

6. Levey AS, Stevens LA, Schmid CH, Zhang YL, Castro AF, Feldman HI, et al. A new equation to estimate glomerular filtration rate. *Ann Intern Med* [Internet]. 2009 [cited 2017 Feb 1];150:604–12. Available from: http://www.ncbi.nlm.nih.gov/pubmed/19414839

7. Wattanakit K, Folsom AR, Selvin E, Weatherley BD, Pankow JS, Brancati FL, et al. Risk factors for peripheral arterial disease incidence in persons with diabetes: the Atherosclerosis Risk in Communities (ARIC) Study. *Atherosclerosis* [Internet]. 2005 [cited 2016 Nov 28];180:389–97. Available from: http://www.ncbi.nlm.nih.gov/pubmed/15910867
