# Supplementary material for: Predictors of sudden cardiac death in atrial fibrillation: The Atherosclerosis Risk in Communities (ARIC) study
Source: PLoS One. 2017 Nov 8;12(11):e0187659. doi: 10.1371/journal.pone.0187659 (PMC5678684; doi:10.1371/journal.pone.0187659)
Supplement: S1 Table — (DOCX) [file pone.0187659.s002.docx]

**S1 Table.** Ethics Committee/Institutional Review Board(s) that approved this study

| National Heart, Lung, and Blood Institute |
| --- |
| University of North Carolina at Chapel Hill, NC |
| Forsyth County Field Center – Wake Forest Baptist Medical Center, Winston-Salem, NC |
| Jackson Field Center - University of Mississippi Medical Center, Jackson, MS |
| Minneapolis Suburbs Field Center – University of Minnesota, Minneapolis, MN |
| Washington County Field Center - Johns Hopkins University, Baltimore, MD |
| Echocardiogram Reading Center - Brigham and Women's Hospital, Boston, MA |
| Atherosclerosis Laboratory - Baylor College of Medicine, Houston, TX |
| Genetics Laboratory - University of Texas Health Science Center, Houston, TX |
| DLCO Reading Center - National Institute of Environmental Health Sciences, Research Triangle Park, NC |
| ECG Reading Center - EPICARE- Wake Forest University Health Sciences, Winston-Salem, NC |
| Pulmonary Function Reading Center – Athens, GA |
| Pulse Wave Velocity Reading Center - University of Texas, Austin, TX |
| ECG Reading Center: Surveillance - University of Minnesota, Minneapolis, MN |
| Clinical Chemistry Laboratory - University of Minnesota, Minneapolis, MN |
| MRI Reading Center - Mayo Clinic, Rochester, MN |
| MRI Vascular Reading Center - Johns Hopkins Hospital, Baltimore, MD |
| Retinal Reading Center - University of Wisconsin, Madison, WI |

Note:

All ARIC data are restricted, requiring prior IRB approval and NHLBI authorization

The data repository is available at the following website:

<https://biolincc.nhlbi.nih.gov/studies/aric/?q=ARIC>

ARIC is a multicenter study and the protocol and informed consent have been reviewed and approved for the IRB at each participating institution.

For the current analysis, the primary IRB is the University of Minnesota IRB which can be contacted at irb@umn.edu.
