# Supplementary material for: Predictors of sudden cardiac death in atrial fibrillation: The Atherosclerosis Risk in Communities (ARIC) study
Source: PLoS One. 2017 Nov 8;12(11):e0187659. doi: 10.1371/journal.pone.0187659 (PMC5678684; doi:10.1371/journal.pone.0187659)
Supplement: S2 Table — (DOCX) [file pone.0187659.s003.docx]

**S2 Table.** Proportional Subdistribution Hazard Ratios (95% Confidence Interval) for SCD and Non-Sudden CV Death in ARIC Participants with Incident AF

|  | # SCD = 110 | | # non-sudden CV deaths = 375 | |
| --- | --- | --- | --- | --- |
| Variables^*^ | HR (95% CI) | P-value | HR (95% CI) | P-value |
| Age (per 5 years) | 1.21 (1.01-1.44) | 0.04 | 1.21 (1.09-1.33) | <0.0001 |
| Men | 1.24 (0.79-1.95) | 0.35 | 0.90 (0.72-1.12) | 0.92 |
| Black race | 1.29 (0.78-2.13) | 0.32 | 1.64 (1.28-2.11) | <0.0001 |
| BMI, kg/m^2^ (per 5 unit increase) | 1.16 (0.96-1.40) | 0.12 | 0.96 (0.87-1.05) | 0.37 |
| Coronary heart disease | 2.59 (1.68-4.00) | <0.0001 | 1.39 (1.08-1.80) | 0.01 |
| Heart failure | 0.82 (0.36-1.84) | 0.63 | 1.57 (1.07-2.29) | 0.02 |
| Hypertension | 1.37 (0.86-2.18) | 0.19 | 1.65 (1.31-2.09) | <0.0001 |
| Diabetes | 1.78 (1.15-2.76) | 0.009 | 1.34 (1.04-1.71) | 0.02 |
| Current smoker | 1.43 (0.90-2.27) | 0.13 | 1.24 (0.96-1.60) | 0.10 |
| eGFR, ml/min/1.73m^2^ (per SD increase) | 1.02 (0.83-1.25) | 0.84 | 1.11 (1.02-1.22) | 0.02 |
| LVH by ECG criteria | 2.18 (1.10-4.34) | 0.03 | 1.34 (0.93-1.93) | 0.12 |
| Heart rate, bpm (per SD increase) | 1.12 (0.93-1.35) | 0.24 | 1.01 (0.91-1.11) | 0.92 |
| QTc interval, ms (per SD increase) | 0.96 (0.47-1.98) | 0.91 | 1.48 (1.02-2.14) | 0.04 |
| HDL, g/dL (per SD decrease) | 1.08 (0.87-1.35) | 0.48 | 1.02 (0.91-1.14) | 0.73 |
| Beta blockers | 1.63 (0.92-2.91) | 0.10 | 1.23 (0.87-1.74) | 0.23 |
| Anti-arrhythmics | 0.85 (0.26-2.84) | 0.79 | 1.19 (0.72-1.95) | 0.50 |
| Digoxin | 1.42 (0.77-2.62) | 0.27 | 1.44 (1.06-1.97) | 0.02 |
| Potassium level (per SD increase) | 0.92 (0.78-1.09) | 0.36 | 1.08 (0.98-1.19) | 0.11 |
| ABI ≤0.9 | 1.01 (0.50-2.02) | 0.99 | 0.90 (0.59-1.37) | 0.63 |
| Albumin, g/dL (per SD decrease) | 1.18 (0.98-1.43) | 0.08 | 1.04 (0.93-1.17) | 0.51 |

ABI indicates ankle brachial index; AF, atrial fibrillation; ARIC, Atherosclerosis Risk in Communities; bpm, beats per minute; dL, deciliter; ECG, electrocardiogram; g, grams; eGFR, estimated glomerular filtration rate; HDL, high density lipoprotein cholesterol; HR, heart rate; LVH, left ventricular hypertrophy; ms, milliseconds; QTc, corrected QT; SCD, sudden cardiac death; SD, standard deviation.
*Variables measured at visit prior to AF diagnosis
