# Supplementary material for: Predictors of sudden cardiac death in atrial fibrillation: The Atherosclerosis Risk in Communities (ARIC) study
Source: PLoS One. 2017 Nov 8;12(11):e0187659. doi: 10.1371/journal.pone.0187659 (PMC5678684; doi:10.1371/journal.pone.0187659)
Supplement: S3 Table — (DOCX) [file pone.0187659.s004.docx]

**S3 Table.** Parsimonious Proportional Subdistribution Hazard Model for Prediction† of SCD and Non-Sudden CV Death in Participants with AF in the ARIC Study

|  | # SCD = 110 | | # non-sudden CV  deaths = 375 | |
| --- | --- | --- | --- | --- |
| Variables | HR (95% CI) | P-value | HR (95% CI) | P-value |
| Age (per 5 years) | 1.16 (0.98-1.37) | 0.09 | 1.20 (1.09-1.32) | <0.0001 |
| BMI (per 5 unit increase) | 1.14 (0.97-1.36) | 0.10 |  |  |
| Coronary heart disease | 2.76 (1.85-4.10) | <0.0001 | 1.40 (1.09-1.79) | 0.008 |
| Hypertension | 1.48 (0.95-2.30) | 0.09 | 1.61 (1.28-2.04) | <0.0001 |
| Diabetes | 2.00 (1.33-3.03) | 0.001 | 1.29 (1.03-1.62) | 0.03 |
| Current smoker | 1.49 (0.97-2.29) | 0.07 | 1.29 (1.01-1.64) | 0.04 |
| LVH by ECG criteria | 2.35 (1.25-4.44) | 0.008 | 1.37 (0.95-1.98) | 0.09 |
| Albumin (per SD decrease) | 1.19 (1.00-1.42) | 0.05 |  |  |
| Race (black) |  |  | 1.63 (1.29-2.08) | <0.0001 |
| Heart failure |  |  | 1.57 (1.07-2.29) | 0.02 |
| Digoxin |  |  | 1.52 (1.13-2.06) | 0.006 |
| eGFR (per SD decrease) |  |  | 1.12 (1.02-1.24) | 0.02 |
| QTc interval (per SD increase) |  |  | 1.50 (1.06-2.12) | 0.02 |

AF indicates atrial fibrillation; ARIC, Atherosclerosis Risk in Communities; bpm, beats per minute; CV, cardiovascular; ECG, electrocardiogram; eGFR, estimated glomerular filtration rate; HR, hazard ratio; LVH, left ventricular hypertrophy; QTc, corrected QT interval; SCD, sudden cardiac death; SD, standard deviation.
† Significant predictors were obtained using backwards elimination (P<0.10) of the candidate predictor variables.
